# Increasing plasma ADAMTS13 activity is associated with HBeAg seroconversion in chronic hepatitis B patients during 5 years of entecavir treatment

**DOI:** 10.1038/s41598-019-42421-5

**Published:** 2019-04-11

**Authors:** Renyong Guo, Yirui Xie, Jiezuan Yang, Haifeng Lu, Ping Ye, Linfeng Jin, Wenqin Lin

**Affiliations:** 1 Department of Laboratory Medicine, First Affiliated Hospital, College of Medicine, Zhejiang University, Key Laboratory of Clinical in Vitro Diagnostic Techniques of Zhejiang Province, Hangzhou, 310003 Zhejiang China; 2 State Key Laboratory for Diagnosis and Treatment of Infectious Diseases, First Affiliated Hospital, College of Medicine, Zhejiang University, Collaborative Innovation Center for Diagnosis and Treatment of Infectious Diseases, Hangzhou, 310003 Zhejiang China; 3 Reproductive Medicine Center, First Affiliated Hospital, College of Medicine, Zhejiang University, Hangzhou, 310003 Zhejiang China

## Abstract

The relationship between hemostatic system and HBeAg seroconversion (SC) of chronic hepatitis B (CHB) patients is ill-defined. We therefore evaluate the predictive value of plasma ADAMTS13 (a disintegrin and metalloproteinase with a thrombospondin motif repeats 13) and VWF (von Willebrand factor) for CHB patients during 5-year entecavir (ETV) treatment. One hundred and fourteen HBeAg positive CHB patients on continuous ETV treatment were recruited. Liver biopsies were evaluated using the METAVIR scoring system, and plasma ADAMTS13 activity (ADAMTS13: AC) and VWF antigen (VWF: Ag) were determined at baseline, 3, 12, 24, 36, and 60 months, respectively. ETV treatment resulted in an increased ADAMTS13: AC and decreased VWF: Ag (both *P* < 0.001) in CHB patients. Cox multivariate analysis demonstrated that the change of ADAMTS13: AC after 1-year ETV treatment was an independent predictor for HBeAg SC at year 5. The area under the receiver operating characteristic (ROC) curve for the change of ADAMTS13: AC after 1-year ETV treatment plus baseline HBV DNA was 0.873 (*P* < 0.001) to predict SC at year 5. The results suggested that increased ADAMTS13: AC after 1 year ETV treatment was associated with a higher seroconversion, and could be used surrogate of HBeAg SC in CHB patients during 5-year ETV treatment.

## Introduction

An estimated 240 million persons worldwide have chronic hepatitis B (CHB) with a varying prevalence geographically, highest in Africa and Asia^1^. The patients with chronic hepatitis B virus (HBV) infection are at risk of developing long-term complications, including liver fibrosis, cirrhosis, liver failure and hepatocellular carcinoma (HCC), which are responsible for over 700,000 deaths annually^2^. Achieving sustained suppression of HBV is an important indicator of immune control of HBV DNA replication and could delay the progression of CHB patients. In the anti- HBV treatment, HBV e antigen (HBeAg) seroconversion (SC) is a critical biomarker for sustaining HBV suppression and postponing disease progression^3,4^.

Clinical data have shown that entecavir (ETV) is one of the most potent nucleoside analogues with a high genetic barrier to resistance and a favorable safety profile during long-term treatment of patients with CHB^5,6^. Compared to lamivudine (LAM), ETV achieved statistically superior virologic, histologic and biochemical improvements in nucleoside-naïve CHB patients^7,8^. Maintained or increased rates of HBV DNA suppression and alanine transferase (ALT) normalization were observed in CHB patients with more than 5 years ETV treatment^9^. These studies have mainly focused on the virological, biochemical and immunological effects of ETV treatment^10,11^, however, few studies have focused on the hemostatic responses of CHB patients during the ETV treatment^12,13^.

Von Willebrand factor (VWF) is synthesized by vascular endothelial cells, and plays a pivotal role in hemostasis. It is released into the plasma as “unusually large” VWF multimers (UL-VWFM) in response to endothelial cell activation or damage. VWF is also produced by transformed sinusoidal endothelial cells following liver injury that is accompanied by necroinflammatory activity^14^. A disintegrin and metalloproteinase with a thrombospondin motif repeats 13 (ADAMTS13) is a metalloproteinase that specifically cleaves multimeric VWF. In the normal state, UL-VWFM is rapidly degraded by ADAMTS13 into smaller VWF multimers, which are less biologically active, and thereby prevent platelet hyper-aggregation and thrombi formation^15^. Nevertheless, ADAMTS13 is significantly reduced in patients with various liver diseases such as hepatic alcoholic hepatitis, liver cirrhosis, veno-occlusive disease^15–17^ and patients undergoing liver transplantation^18^. ADAMTS13 deficiency increases plasma UL-VWFM levels, which results in increased platelet aggregation and trending to platelet thrombi under high shear stress, and may cause sinusoidal microcirculatory disturbances that promote liver disease progression^14,19^.

In this study, we examined plasma levels of ADAMTS13 activity (ADAMTS13: AC), and VWF antigen (VWF: Ag) in serial samples of patients with HBeAg positive (HBeAg+) CHB who underwent up to 5 years of ETV treatment. The aim of the study was to determine the kinetic profile of plasma ADAMTS13: AC and VWF: Ag of HBeAg+ CHB patients during 5 years of ETV treatment, and to identify that the factors could be used to predict HBeAg SC of patients subjecting to long-term antiviral treatment. Furthermore, this result would contribute to determine that ADAMTS13 be a target for treating patients with chronic HBV infection^15^.

## Materials and Methods

### Study population

One hundred and fifty two consecutively ETV treatment-naïve CHB patients were enrolled in this study from the Department of Infectious Diseases of the First Affiliated Hospital of Zhejiang University (Hangzhou, China) from February 2008 to May 2015. CHB diagnosis was made according to the diagnostic standards of the Asian-Pacific clinical practice guidelines on the management of hepatitis B^20^. Patients were treated with oral ETV (0.5 mg daily), and the enrolling criteria for patients were also referred our previously reports^21,22^. In addition, the exclusion criteria for CHB patients enrolled was (1) co-infection with either hepatitis A, hepatitis C, or hepatitis D viruses or human immunodeficiency virus (HIV); (2) alcohol- or drug-induced autoimmune liver diseases, other acquired or inherited causes of liver disease, liver cirrhosis; (3) diabetes mellitus or thromboembolic diseases, or other severe diseases. Healthy controls (n = 88) with normal serum alanine aminotransferase (ALT) level and undetectable HBsAg and other serological markers of hepatitis B were also registered from the Health Examination Center of the First Affiliated Hospital of Zhejiang University, and were matched for age and gender with CHB patients.

Patient’s follow-up and blood drawn were performed at 3-month intervals during the first year and 6-month intervals thereafter for clinical assessments including liver biochemical tests, hematologic examinations, measurements of serological hepatitis B markers and HBV DNA levels. The follow-up period for each patient began at the time of ETV treatment, and the time point was recorded at the occurrence of HBeAg SC. HBeAg SC defined as the loss of HBeAg and presence of anti-HBe antibody (HBeAb) in peripheral blood. This study was conducted in agreement with the ethical principles of the Declaration of Helsinki, and the study protocol was approved by the Ethics Committee of the First Affiliated Hospital of Zhejiang University. Moreover, informed consent was obtained from each subject prior to enrollment.

### Liver biopsy

All the 152 CHB patients were subjected to a liver biopsy before ETV treatment. Liver biopsy was performed under the guidance of ultrasound using a second rapid biopsy puncture to obtain liver tissue, and the length of liver tissue was about 15~20 mm, formalin fixed, paraffin embedded, serial sectioned, stained using HE staining. The stained slides were observed and diagnosed by two pathologists, respectively, under an optical microscope. Liver fibrosis was staged according to the METAVIR scoring system as following: No fibrosis (F0), mild fibrosis (portal fibrosis without septa, F1), moderate fibrosis (portal fibrosis with few septa, F2), severe fibrosis (numerous septa without cirrhosis, F3) or cirrhosis (F4). The more detail process was shown in previous report^23^.

### Laboratory assays

Fasting blood samples were obtained from an antecubital vein, the whole blood, anti-coagulated with ethylenediaminetetraacetic acid (EDTA) (2.4 mg per 2 ml venous blood), was used for the immediate white blood cell (WBC) and platelet (PLT) counts. The anticoagulant plasma was immediately isolated by centrifuging the whole blood in a refrigerated centrifuge at 1500 × *g* for 10 min and stored at −80 °C until assay. Levels of serum albumin (ALB), ALT, aspartate transaminase (AST), and total bilirubin (TB) were determined using a Hitachi 7600 analyzer (Hitachi Ltd., Tokyo, Japan). Serologic tests for HBsAg, HBeAg, anti-HBs antibody, and anti-HBe antibody were conducted by enzyme immunoassay (AxSYM; Abbott Laboratories, Abbott Park, IL, USA). Plasma VWF: Ag and ADAMTS13: AC were determined using commercial enzyme-linked immunosorbent assay (ELISA) kits (R&D Systems Inc., Minneapolis, MN, USA) according to the manufacturer’s instructions. Plasma HBV DNA levels were assayed using the COBAS TaqMan HBV test (Roche Diagnostics, Indianapolis, IN, USA) as our previous report^22^.

The status of liver fibrosis were also determined by non-invasive methods of FIB-4 index, calculated using the formula^24^: FIB-4 = Age (yr) × AST (U/L)/[PLT account (10^9^/L) × ALT^1/2^ (U/L)].

### Statistical analysis

Continuous variables are expressed as mean ± standard deviation (SD). Serum HBV DNA and HBsAg levels are expressed in logarithmic scale (log10). The comparison of continuous data was performed using Student’s t test or Mann–Whitney U test for two independent groups and a paired t test for two related groups. Categorical variables were compared using Pearson’s chi-squared test or Fisher’s exact test as appropriate. Spearman rank correlation was used to evaluate the relationship between parameters. To predict treatment outcome, cut-off points for continuous variables were determined by receiver operating characteristic (ROC) curve analysis. Factors attaining a *P*-value of less than 0.1 in univariate analysis were evaluated by multivariate analysis using Cox proportional hazard regression analyses. Kaplan-Meier curves were used for the estimation of outcome rates over time. *P* < 0.05 was considered statistically significant. All statistical tests were performed using SPSS, version 19.0 (SPSS Inc., Chicago, IL, USA).

## Results

There was a significant decline in HBsAg levels from baseline to month 60 after commencing ETV (4.1 vs. 3.1, log10 IU/mL, *P* = 0.002) in all enrolled CHB patients (HBeAg SC and non-SC). However, none of the patients experienced clearance of HBsAg during the 5-year of ETV treatment. Additionally, no other liver-related complications occurred during ETV treatment, no serious adverse events were identified or hepatitis flares were observed throughout the entire 5-year ETV treatment. The minor complications included mild fever and rash, which could be quickly recovered with symptomatic treatment.

### Clinical data of CHB patients during the 5 years of ETV treatment

**Figure 1:**
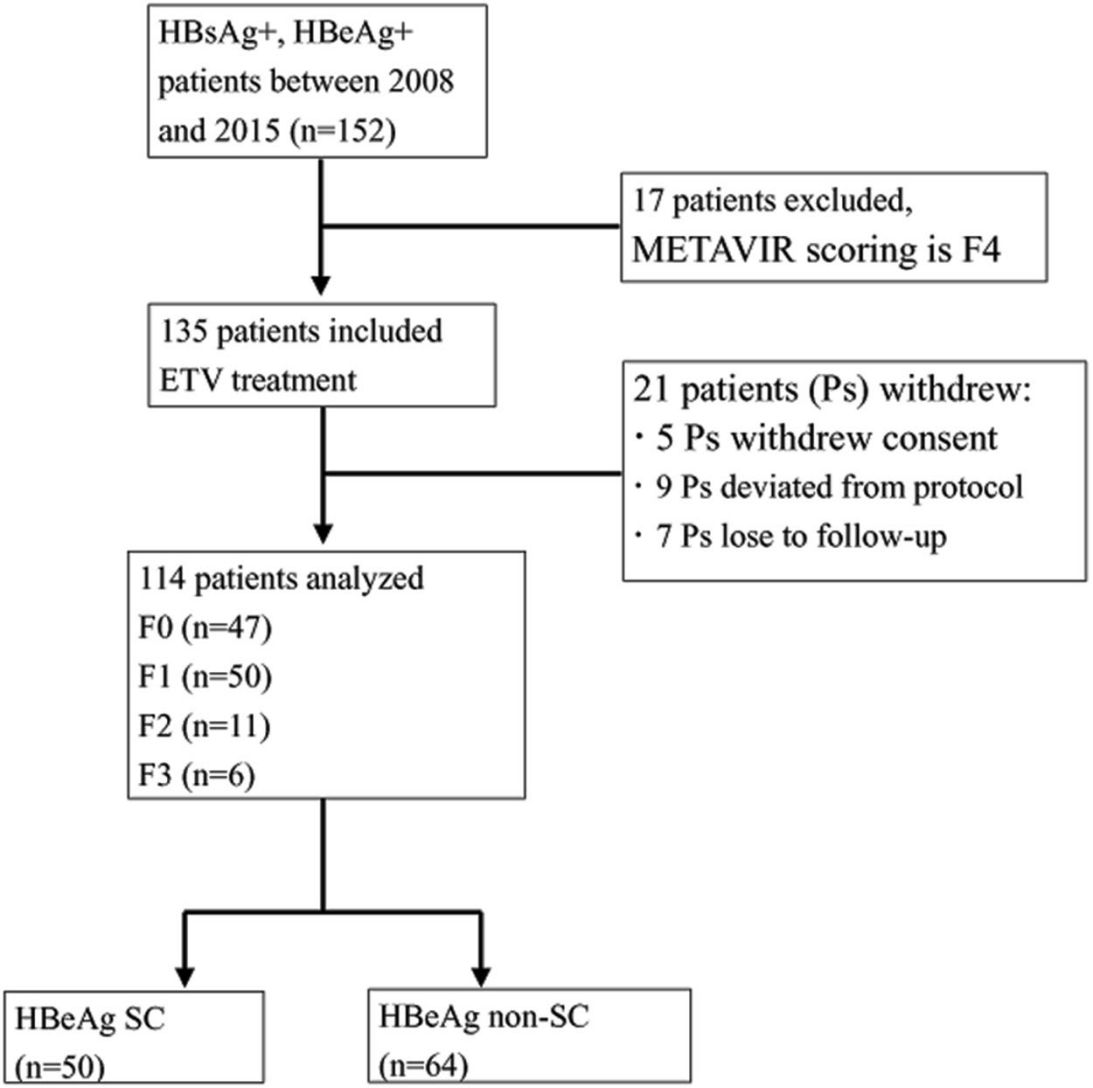
Flowchart of the CHB patients included in the study. Liver fibrosis stage (F0~F4) is according to the METAVIR scoring system.

According to the results of liver biopsy, the 135 CHB patients (17 patients with cirrhosis excluded, METAVIR scoring is F4) were enrolled for 5-year ETV treatment. Finally, a total of 114 CHB patients (114/135, 84.4%) completed 5-year ETV treatment and 21 patients (15.6%) withdrew prematurely. The more detail was shown in Fig. 1. Higher baseline levels of FIB-4, ALT, AST, TB, and VWF: Ag, along with lower levels of ALB, PLT and ADAMTS13: AC were found in patients compared with healthy controls. Additionally, Levels of FIB-4, ALT, AST, TB, HBsAg, HBeAg, and VWF: Ag significantly decreased after 5-year ETV treatment, whereas increased levels of PLT and ADAMTS13: AC were detected after treatment. Concurrently, HBV was effectively suppressed in CHB patients during ETV treatment, and all patients reached undetectable HBV DNA (<20 IU/mL) after 5-year ETV treatment. The clinical profile of the enrolled CHB patients and healthy controls are shown in Table 1.

**Table 1:** Clinical and biochemical characteristics of patients with chronic hepatitis B (CHB) before and after 5 years of entecavir treatment.

Variable	CHB (n = 114)		Healthy control (n = 88)	*p*-values^a^	*p*-values^b^
	Baseline	Year 5			
Sex (male/female)	84/30	—	59/29	0.304^c^	NA
Age (years)	35.1 ± 9.6	—	36.4 ± 10.5	0.870	NA
HBV genotype (B/C/D)	53/44/17	—	—	NA	NA
Metavir score (F0–F1/F2–F3)	97/17	—	—	NA	NA
FIB-4	2.15 ± 1.22	1.07 ± 0.51	0.87 ± 0.37	0.000	0.016
ALT (U/L)	200.5 ± 102.2	21.7 ± 7.3	19.6 ± 6.27	0.000	0.000
AST (U/L)	126.0 ± 63.6	19.2 ± 7.8	20.7 ± 6.81	0.000	0.000
TB (umol/L)	15.6 ± 6.1	10.8 ± 3.8	10.1 ± 3.3	0.001	0.005
ALB (g/L)	45.6 ± 5.5	45.9 ± 5.7	48.1 ± 2.5	0.012	0.692
WBC (×10^9^/L)	4.8 ± 1.2	5.4 ± 1.2	5.7 ± 1.1	0.058	0.082
PLT (×10^9^/L)	159.1 ± 57.8	190.9 ± 45.9	214.1 ± 57.7	0.001	0.004
HBV DNA (log_10_ IU/mL)	7.5 ± 1.6	Undetectable	Undetectable	NA	NA
HBsAg (log_10_ IU/mL)	4.1 ± 0.8	3.1 ± 0.6	Undetectable	NA	0.002
HBeAg (PEIU/mL)	126.5 ± 65.5	0.74 ± 0.4	Undetectable	NA	0.000
VWF: Ag (U/L)	1491.4 ± 224.4	1060.4 ± 224.8	1026.4 ± 255.2	0.000	0.000
ADAMTS13: AC (U/mL)	815.4 ± 204.4	1341.4 ± 267.6	1669.2 ± 370.9	0.000	0.000

Values expressed as mean ± standard deviation, unless otherwise indicated; VWF: Ag, VWF antigen; ADAMTS13: AC, ADAMTS13 activity; NA, not available.

^a^*p*-values for differences between CHB baseline and healthy control are from Mann-Whitney *U*-test.

^b^*p*-values for differences between CHB baseline and CHB year 5 are from two-tailed paired t tests.

^c^*p*-values are from Chi-square test.

### Relationship between the ADAMTS13: AC and clinical parameters

**Figure 2:**
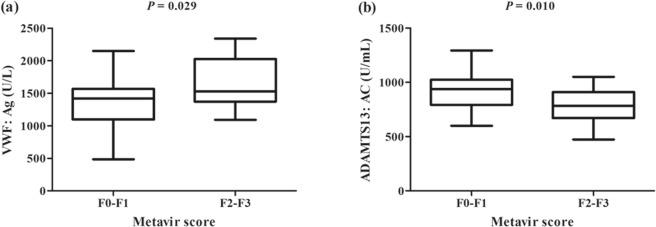
Mann–Whitney analysis of baseline levels of VWF: Ag and ADAMTS13: AC stratified by METAVIR score.

The ADAMTS13: AC levels at baseline correlated positively with ALB and PLT, and correlated negatively with FIB-4, ALT and HBV DNA (*P* < 0.05, all). Additionally, there was no significantly relationship between the plasma levels of ADAMTS13: AC and VWF: Ag at baseline (Table 2). Moreover, the relationships between Metavir score and plasma levels of ADAMTS13: AC and VWF: Ag was also analyzed. As shown in Fig. 2, patients with Metavir score F2-F3 exhibited higher levels of VWF: Ag and lower levels of ADAMTS13: AC at baseline than patients with Metavir score F0-F1 (both *P* < 0.05).

**Table 2:** Correlation coefficients between the baseline levels of ADAMTS13: AC and clinical variables.

Variables	Correlation coefficients^a^	*p*-values
Age (years)	−0.201	0.199
FIB-4	−0.396	0.012
ALT (U/L)	−0.349	0.033
AST (U/L)	−0.246	0.152
TB (umol/L)	−0.287	0.117
ALB (g/L)	0.322	0.042
WBC (×10^9^/L)	−0.135	0.340
PLT (×10^9^/L)	0.343	0.036
HBV DNA (log_10_ IU/mL)	−0.421	0.004
HBsAg (log_10_ IU/mL)	−0.294	0.102
HBeAg (PEIU/mL)	−0.305	0.093
VWF: Ag (U/L)	−0.317	0.056

^a^Spearman’s rank correlation coefficient.

### Dynamic changes of clinical parameters during antiviral treatment

After 5-year ETV treatment, 43.9% of patients (50 of 114) underwent HBeAg SC. The non-SC HBeAg patients (n = 64) had significantly higher baseline levels of AST, HBV DNA, HBsAg, and HBeAg than that of SC group (n = 50). Furthermore, decrease in FIB-4, ALT, AST, TB, HBV DNA, HBsAg, HBeAg, and VWF: Ag, along with increase in PLT and ADAMTS13: AC after 5-year treatment were detected in both groups (data no shown).

The changes in HBsAg (ΔHBsAg, −1.1 vs. −0.8 log_10_ IU/mL, *P* = 0.009) and HBeAg (ΔHBeAg, −137.0 vs. −65.5 Paul Ehrlich Institute Units (PEIU)/mL, *P* = 0.005) were significantly higher in non-SC patients than that of HBeAg SC group after 1-year ETV treatment. In contrast, change in ADAMTS13: AC (ΔADAMTS13: AC, 361.4 U/mL vs. 107.6 U/mL, *P* = 0.003) in the HBeAg SC group was significantly higher than that in non-SC group. Moreover, after 5-year ETV treatment, ΔHBsAg (−1.2 vs. −0.7 log_10_ IU/mL, *P* = 0.021) and ΔHBeAg (−167.5 vs. −73.6 PEIU/mL, *P* = 0.002) were also significantly higher in non-SC patients than SC group (Table 3).

**Table 3:** Comparisons of parameters in CHB patients with and without HBeAg seroconversion during 5-year entecavir treatment.

Variable	Non- HBeAg seroconversion (n = 64)			HBeAg seroconversion (n = 50)			*p*-values^a^	*p*-values^b^	*p*-values^c^
	Baseline	Δ value 1	Δ value 2	Baseline	Δ value 1	Δ value 2			
Sex (male/female)	48/16	—	—	36/14	—	—	0.718^d^	—	—
Age (years)	35.6 ± 9.1	—	—	34.6 ± 9.9	—	—	0.424	—	—
HBV genotype (B/C/D)	31/25/8	—	—	22/19/9	—	—	0.706^d^	—	—
Metavir score (F2–F3/F0–F1)	12/52	—	—	5/45	—	—	0.150^d^	—	—
FIB-4	2.47 ± 2.24	−1.06 ± 0.67	−1.24 ± 0.86	1.95 ± 1.26	−0.78 ± 0.84	−0.93 ± 1.24	0.156	0.183	0.148
ALT (U/L)	222.7 ± 104.7	−198.8 ± 143.3	−199.5 ± 147.2	172.2 ± 92.5	−152.3 ± 98.5	−155.1 ± 110.5	0.104	0.163	0.197
AST (U/L)	142.3 ± 67.1	−122.4 ± 81.5	−123.0 ± 79.4	105.1 ± 52.4	−84.8 ± 72.7	−86.7 ± 69.9	0.041	0.068	0.034
TB (umol/L)	15.8 ± 6.3	−3.5 ± 7.7	−6.5 ± 9.2	15.2 ± 5.5	−1.7 ± 4.7	−4.9 ± 6.9	0.713	0.645	0.607
ALB (g/L)	45.2 ± 5.5	1.0 ± 2.4	0.4 ± 3.3	46.0 ± 5.6	−0.1 ± 2.2	0.1 ± 2.6	0.119	0.103	0.677
WBC (×10^9^/L)	4.9 ± 1.2	0.7 ± 1.2	0.8 ± 1.3	4.7 ± 1.1	0.3 ± 1.4	0.4 ± 1.5	0.672	0.414	0.338
PLT (×10^9^/L)	152.7 ± 54.2	39.8 ± 46.4	43.5 ± 40.9	167.2 ± 61.8	35.7 ± 39.1	38.0 ± 38.5	0.172	0.538	0.679
HBV DNA (log_10_ IU/mL)	7.9 ± 1.8	−5.4 ± 0.5^e^	—	6.9 ± 1.2	−4.7 ± 0.5^f^	—	0.001	0.482	—
HBsAg (log_10_ IU/mL)	4.4 ± 0.7	−1.1 ± 0.5	−1.2 ± 0.7	3.6 ± 0.6	−0.8 ± 1.1	−0.7 ± 0.8	0.000	0.009	0.021
HBeAg (PEIU/mL)	167.0 ± 58.9	−137.0 ± 88.7	−167.5 ± 91.2	74.7 ± 23.8	−65.5 ± 85.0	−73.6 ± 102.4	0.002	0.005	0.002
VWF: Ag (U/L)	1501.9 ± 279.0	−135.0 ± 459.0	−432.0 ± 504.0	1477.9 ± 193.1	−292.5 ± 441.0	−405.0 ± 747.0	0.579	0.224	0.478
ADAMTS13: AC (U/mL)	789.5 ± 209.3	107.6 ± 276.1	444.6 ± 456.5	848.5 ± 194.9	361.4 ± 298.9	680.9 ± 620.9	0.096	0.003	0.275

Values expressed as mean ± standard deviation, unless otherwise indicated; △value 1, differences in values at baseline and at year 1 post-treatment. Δ value 2, differences in values at baseline and at year 5 post-treatment.

^a^*p*-values for differences between with and without HBeAg seroconversion of CHB patients at baseline, ^b^*p*-values for at year 1 post-treatment (Δ value 1), ^c^*p*-values for at year 5 post-treatment (Δ value 2), are all from Mann-Whitney *U*-test. ^d^*p*-values are from Chi-square test. ^e^values are from 21 patients with HBV DNA ≥ 20 IU/mL at year 1. ^f^values are from 5 patients with HBV DNA ≥ 20 IU/mL at year 1.

**Figure 3:**
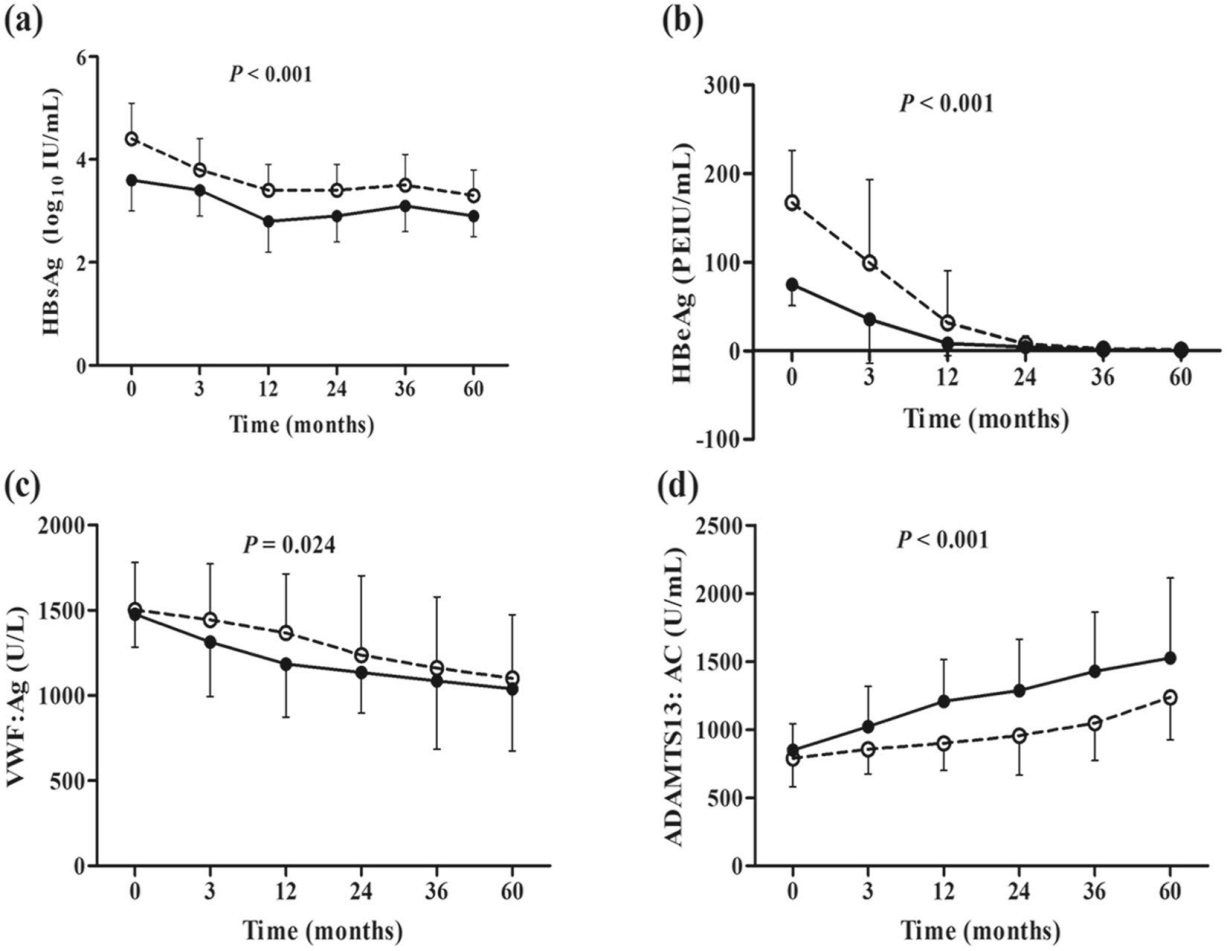
Dynamic profile of clinical parameters in CHB patients during 5-year entecavir treatment. The respective *P*-values refer to the difference between the patients with or without HBeAg seroconversion (SC), evaluated by repeated measurement analysis. Open circles, HBeAg non-SC; full circles, HBeAg SC.

The dynamic changes in several laboratory parameters of the two groups from baseline through post-treatment are shown in Fig. 3. The changes of HBsAg, HBeAg, VWF: Ag, and ADAMTS13: AC showed significant difference between HBeAg SC and non-SC groups (*P* < 0.05, all).

### Predictive values of clinical parameters after 1 year of ETV treatment

Cox univariate regression analysis demonstrated that those patients with lower baseline HBV DNA levels, but higher rate of virologic response and higher ΔADAMTS13: AC after 1 year of ETV treatment had significantly greater probability of HBeAg SC (*P* < 0.05, all). Based on multivariate analysis, the baseline HBV DNA levels (Hazard ratio [HR] = 0.723, 95% confidence interval [CI] = 0.514–0.976, *P* = 0.043) and ΔADAMTS13: AC levels after 1 year of ETV treatment (HR = 1.109, 95% CI = 1.021–1.185, *P* = 0.015) were independent predictors of HBeAg SC at year 5 (Table 4).

**Table 4:** Cox regression analyses of changes of clinical parameters in CHB patients after 1 year of entecavir treatment for prediction of HBeAg seroconversion at year 5.

Variables	HR (95% CI)	*p*-values
Univariate analysis		
Sex (female vs. male)^a^	0.972 (0.390–2.422)	0.952
Age^a^	0.996 (0.957–1.038)	0.855
HBV genotype (non-C vs. C)^a^	0.710 (0.338–1.494)	0.367
Metavir score (F2-F3/F0-F1)^a^	0.551 (0.165–1.834)	0.331
FIB-4	1.282 (0.711–2.310)	0.409
ALT	1.002 (0.999–1.005)	0.128
AST	1.004 (0.999–1.101)	0.152
TB	1.037 (0.971–1.107)	0.274
ALB	0.861 (0.721–1.027)	0.096
WBC	0.900 (0.649–1.249)	0.529
HBsAg	1.503 (0.850–2.652)	0.160
HBeAg	1.005 (1.000–1.010)	0.057
HBV DNA^a^	0.620 (0.459–0.838)	0.002
Virologic response (Y/N)^b^	3.580 (1.074–11.941)	0.038
VWF: Ag	0.997 (0.947–1.042)	0.116
ADAMTS13: AC	1.115 (1.052–1.181)	0.006
Multivariate analysis		
ALB	1.044 (0.914–1.192)	0.527
HBeAg	1.001 (0.996–1.006)	0.704
HBV DNA^a^	0.723 (0.514–0.976)	0.043
Virologic response (Y/N)	1.079 (0.180–6.471)	0.933
ADAMTS13: AC	1.109 (1.021–1.185)	0.015

^a^Clinical characteristic before entecavir (ETV) therapy (at baseline); HR: hazard ratio; CI: confidence interval. ^b^Undetectable level of serum HBV DNA after 1 year of ETV treatment. Y: yes, N: no.

**Figure 4:**
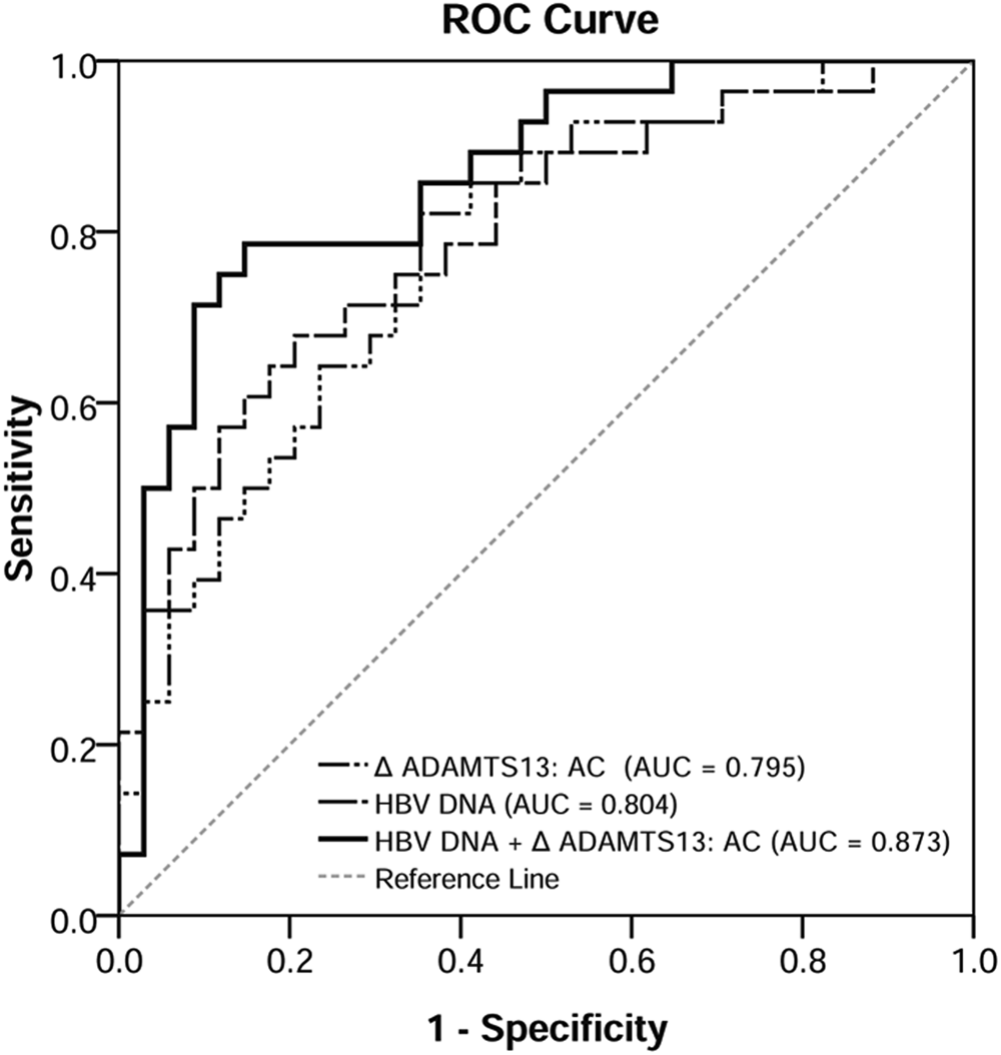
Receiver operator characteristic (ROC) curves. The ROC curves of the pre-treatment levels of HBV DNA, ΔADAMTS13: AC after 1 year of ETV treatment, and their combination for differentiating the year 5 HBeAg SC in HBeAg positive CHB patients.

We further performed ROC analysis to evaluate the ability of pre-treatment levels of HBV DNA and ΔADAMTS13: AC levels after 1 year of ETV treatment for predicting a year 5 HBeAg SC. The AUC values were consistently high for HBV DNA (AUC = 0.804, 95% CI = 0.683–0.930) and ΔADAMTS13: AC (AUC = 0.795, 95% CI = 0.646–0.908), whereas no differences were observed between them. When ΔADAMTS13 level was combined with HBV DNA, the AUC of HBV DNA + ΔADAMTS13: AC was 0.873 (95% CI = 0.785–0.959). The AUC of them was higher than ΔADAMTS13: AC or HBV DNA alone (both *P* < 0.05) (Fig. 4).

**Figure 5:**
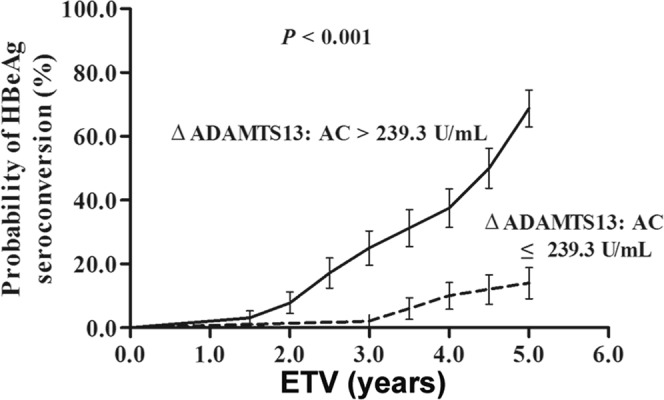
Probability of HBeAg SC related to the change of ADAMTS13: AC in 114 HBeAg positive CHB patients after 1 year ETV treatment (*P* < 0.001 by log-rank test).

Furthermore, when the cut-off value of 239.3 U/mL for ΔADAMTS13: AC after 1 year of ETV treatment were applied to assess the cumulative rates of HBeAg SC, patients with ΔADAMTS13:AC > 239.3 U/mL (HR = 6.618, 95% CI = 2.977–14.714, *P* < 0.001) were found to achieve significantly higher probability of HBeAg SC (Fig. 5).

## Discussion

In this study, we report a dynamic relationship between plasma VWF: Ag, ADAMTS13: AC levels and HBeAg SC in HBeAg + CHB patients during 5-year ETV therapy. The degree of plasma ADAMTS13: AC increased after 1 year of ETV treatment was highly predictive of HBeAg SC in CHB patients. To the best of our knowledge, the present study is the first report to describe the dynamic change of VWF: Ag and ADAMTS13: AC in CHB patients undergoing 5 years of ETV treatment. Moreover, the result indicated that the change in ADAMTS13: AC levels help to predict the clinical outcome of CHB patients during ETV treatment with the predictive cutoff value of 239.3 U/ml.

It is reported that the hemostasis experiences rebalancing process in chronic liver disease, but the balance is fragile and may be perturbed by many disease, such as infection or renal failure^25^. Severe liver diseases are accompanied by multiple changes in the hemostatic system, which may lead to microcirculatory disturbances and involving in hepatic parenchymal extinction, the acceleration of liver fibrosis and disease deterioration^26,27^. It was reported that plasma ADAMTS13: AC concomitantly decreased with functional liver capacity and was closely related to the severity of liver disturbance^15,28^. Likewise, Hugenholtz GC *et al*. found that decreasing ADAMTS13: AC was associated with poor outcome in patients with acute liver failure as evidenced by higher grades of encephalopathy, higher transplantation rates, and lower survival^18^. The relationship between pre-treatment levels of ADAMTS13: AC and baseline ALT or ALB in our study suggests that the severity of hepatic impairment be associated with severity of ADAMTS13 deficiency. The negative correlation between levels of ADAMTS13: AC and HBV DNA in our study further indicated that highly replicative activity of HBV might be an important factor affecting the synthetic ability of ADAMTS13 in liver. Additionally, negative association was observed between plasma ADAMTS13: AC and liver fibrosis stage in the present study, consistent with prior observations that plasma ADAMTS13: AC was related to developing various complications and survival time in patients with liver cirrhosis^29,30^.

Evaluating the outcome of antiviral therapy using ADAMTS13 may help assess the microcirculatory disturbances caused by imbalanced levels of VWF and ADAMTS13, because level of VWF, the substrate of ADAMTS13, plays an essential role in primary hemostasis and is progressively increased as functional liver capacity decreases^16,19^. Decreased ADAMTS13: AC in CHB patients indicates declining capacity to degrade large VWF molecules^29,30^. In the study, the plasma VWF: Ag levels of CHB patients were higher than that of healthy control at baseline. Although no significant difference of VWF: Ag was found between with and without HBeAg SC patients at each follow-up time point during ETV treatment, interestingly, changes over time in VWF: Ag differed significantly between patients with and without HBeAg SC (Fig. 3c). Similarly, it was reported that VWF can serve as a biomarker, and perhaps an alternative target for therapeutic intervention of HCC progression and HBV viral infection^31^.

The significant decreases in ADAMTS13: AC and increases in VWF: Ag suggested that hyper-coagulable and consumptive states exist in CHB patients^29^. The ADAMTS13 levels were possibly affected simultaneously by decreased hepatic production and increased consumption due to cleavage of UL-VWFM^13,30^. Although a compensatory mechanism in which patients with higher VWF levels increase hepatic ADAMTS13 production to maintain the balance between VWF and ADAMTS13 maybe enrolled^19^. At the present study, ADAMTS13: AC levels were consistently lower in patients without HBeAg SC than in those with SC throughout the treatment, indicating limited compensatory ability for ADAMTS13 synthesis in liver. Meanwhile, the negative relationship between ADAMTS13: AC and the HBV DNA load in our study suggests that persistent HBV infection may lead to declining ability of hepatic ADAMTS13 synthesis. Damaged liver function with higher AST and replication of HBV in patients without HBeAg SC might affect ADAMTS13 synthesis to a greater extent. Recently evidence from epidemiological, animal and clinical studies indicates that intrahepatic activation of hemostasis and microcirculatory disturbance contributes to liver disease progression^32–34^. We hypothesize that imbalance between ADAMTS13 and VWF in the hepatic sinusoid might induce formation of platelet thrombi within the hepatic microvasculature, which led to tissue ischemia and hepatic dysfunction resulting in the progression of CHB in patients with low ADAMTS13: AC^35^. However, the exact mechanisms and factors that contribute to this relationship remain to be clarified.

There are some limitations in our study. Even though patients were prospectively followed, it is a retrospective study with a limited number of patients. Moreover, the determination of VWF: Ag concentration was unable to provide specific information regarding the activity of circulating VWF molecular species. A deeper analysis of VWF propeptide, VWF multimers and the activity of VWF might better clarify the mechanisms underlying our findings. In addition, mutation of the ADAMTS13 gene, marked cytokinemia, enhanced endotoxemia and/or the presence of protease inhibitors may be closely related to declining ADAMTS13: AC^14,17^. It is necessary to involve these factors into the measurement and analysis of VWF and ADAMTS13 in future study. Furthermore, It would be recommended to stratify and analyze the relationship between the progression of liver disturbances and ADAMTS13: AC or VWF: Ag in CHB patients during different stage of antiviral therapy. In addition, it will be interesting to determine and analyze the relationship among plasma ADAMTS13, VWF and IP-10 in future study.

In conclusion, our results indicate that the changes in ADAMTS13: AC after 1 year of ETV treatment is associated with HBeAg SC of CHB patients during 5-year ETV treatment. Monitor of ADAMTS13: AC during ETV treatment could provide utility for early prediction of progressive liver disease and may help to predict long-term HBeAg SC in patients with CHB. Moreover, the presented results raise the possibility of novel supportive therapies of ADAMTS13 supplementation for improving intrahepatic microcirculatory disturbance and outcomes of CHB patients, although, more cases should be required and further investigated.
